# An evaluation of serial blood lactate measurement as an early predictor of shock and its outcome in patients of trauma or sepsis

**DOI:** 10.4103/0972-5229.56051

**Published:** 2009

**Authors:** Uma Krishna, Suresh P. Joshi, Mukesh Modh

**Affiliations:** Department of Surgery, Choithram Hospital and Research Centre, Indore, M.P, India; 1 Dept. of Pathology, Choithram Hospital and Research Centre, Indore, India; 2 Choithram Hospital and Research Centre, Indore, India

**Keywords:** Outcome, sepsis, serial lactate values, shock, trauma

## Abstract

**Context:**

Attainment of hemodynamic parameters to within a normal range may leave patients in compensatory shock. In such patients, serial blood lactate evaluation can be useful in predicting shock.

**Aims:**

To ascertain the role of serum lactate as a predictor of shock and its outcome in patients of trauma and sepsis.

**Settings and Design:**

A prospective, non-interventional study.

**Materials and Methods:**

The study included 50 patients (5 to 60 years old) of trauma admitted within 12 hours of injury and patients of suspected or proven sepsis. Those with chronic medical illnesses, alcohol intoxication, or poisoning were excluded. Blood lactate levels were analyzed at admission and 12, 24, and 36 hours of inclusion with records of corresponding hemodynamic variables, investigations, and interventions. The outcome was recorded as survival or non-survival.

**Statistical Analysis Used:**

Statistical analysis was done with a student's t test and repeated measure ANOVA (Analysis of Variance).

**Results:**

An analysis revealed higher mean lactate levels in non-survivors as compared with survivors. Mean lactate levels in non-survivors did not attain normal levels, while that of survivors reached normal levels by 24 hrs in trauma patients and 36 hrs in sepsis patients. The predicted mortality rates by a lactate level >40 mg/dl at admission, 12, 24, and 36 hours were 52.6%, 61.5%, 83.3%, and 100%, respectively for both the subgroups combined. Non-survivors had a higher incidence of MODS (Multi Organ Dysfunction Syndrome).

**Conclusions:**

Serial lactate values followed over a period of time can be used to predict impending complications or grave outcome in patients of trauma or sepsis. Interventions that decrease lactate values to normal early may improve chances of survival and can be considered effective therapy. Lactate values need to be followed for a longer period of time in critical patients.

## Introduction

The arrival of a patient of trauma sets a whole team of doctors and nurses into a flurry of well-directed activity. The patient gains a new individuality as a body system under intensive and invasive monitoring. The cascade of events initiated by the traumatic impact, the Systemic Inflammatory Response Syndrome (SIRS), is also ongoing in a patient of sepsis. This continuum of clinical and pathophysiological events triggered in the body is manifested as shock. If undeterred, it extends to the ominous Multi Organ Dysfunction Syndrome (MODS). Serial blood lactate evaluation can be useful in predicting shock in patients in the compensated stage with normal hemodynamic parameters. Elevated blood lactate levels provide an insight into the presence of global tissue hypoxia – a forerunner to the development of shock and MODS. Timely identification of ongoing events before they take an ominous turn is essential in the management of the patient in shock.[1–3]

## Materials and Methods

### Study Design

From October 2003 to 2004, a prospective, non-interventional study was undertaken after ethical committee approval in the Department of Surgery, at the Choithram Hospital and Research Centre in Indore, M.P., India. A total of 50 patients were included in the study.

### Inclusion and Exclusion Criteria

The study included the following patients, in the age group of 5-60 years old, in the Intensive Care Unit (ICU) or ward:

- Patients admitted within 12 hrs of trauma including road accidents, burns, railroad accidents, fall from height and assault, etc.
- Patients of suspected or overt sepsis including those meeting criteria for SIRS, septic shock, and MODS

Patients with the following positive history were excluded from the study:

- Co morbidities - bronchial asthma, diabetes, ischemic heart disease, congestive heart failure, renal failure, renal transplant, malignancy, chronic pancreatitis
- History of acute alcohol ingestion, ingestion of poison
- Chronic medication for diabetes, asthma, tuberculosis, iron supplementation, epilepsy, AIDS
- Known inborn error of lactate metabolism
- The patients were admitted and treated as deemed necessary under different surgical units.

### Data Collection

The following data was collected:

- Hospital registration number
- Date and time of injury/inclusion into study
- Vitals on admission and at regular intervals with records of hourly urine output, oxygen saturation, and central venous pressure (CVP), as and when available.
- Blood lactate levels at admission, 12 hrs, 24 hrs, and 36 hrs.
- Initial work-up: hemoglobin, packed cell volume, total and differential WBC counts, random blood sugar, serum electrolytes, arterial blood gases (as per discretion of the treating doctor)
- Documentation of organ dysfunction with serum creatinine, serum bilirubin, platelet count, chest X-ray, arterial blood gases (where available)
- The outcome was recorded as survival or non-survival
- A record of the number of days of hospital stay was also kept after inclusion into the study

Treatment was left to the discretion of the attending consultant.

Finally, records were also kept of the types of organ dysfunction and certain interventions including ventilator support (V), dialysis (D), and surgery.

### Sample Collection and Analysis

A total of 5 ml of heparinized venous blood was collected without stasis in vacuum containers with fluoride as a reagent (to inhibit glycolysis) and transported to the laboratory for analysis. Storage, if necessary, was done in a closed container at 4-6°C.

The analysis of plasma lactate was carried out by the commercial kit supplied by Randox (UK) on a semi-automated system 5010 using corresponding standards and quality control sera. The method of determination is based on an enzymatic conversion of lactate to pyruvate and hydrogen peroxide. The hydrogen peroxide is converted to pyruvate and nascent oxygen. This nascent oxygen then oxidizes 4-amino-phenazone to a colored compound, which is measured colorimetrically.[4]

### Statistical Analysis

Statistical analysis was done using a t-test as well as Repeated Measures ANOVA (SPSS 14 Statistics Package). A *P*-value less than 0.05 was taken as statistically significant.

## Results

**Figure 1:**
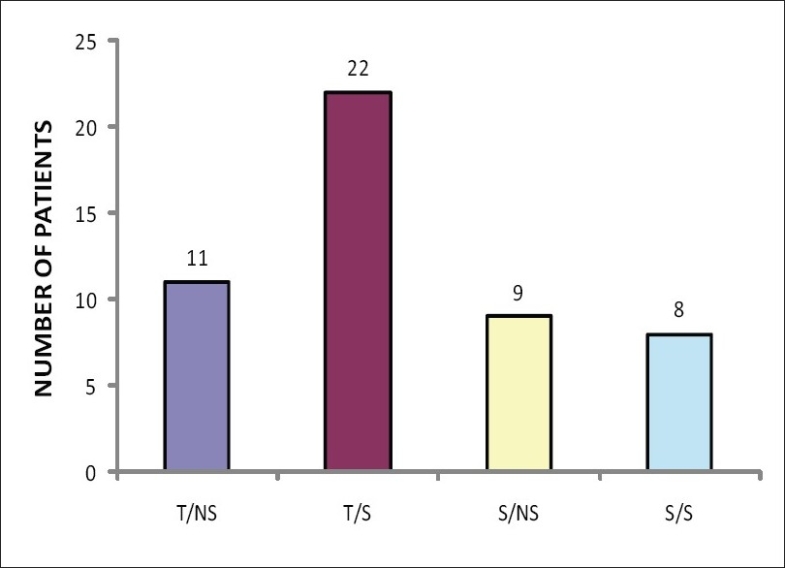
Distribution of patients of trauma and sepsis

**Figure 2:**
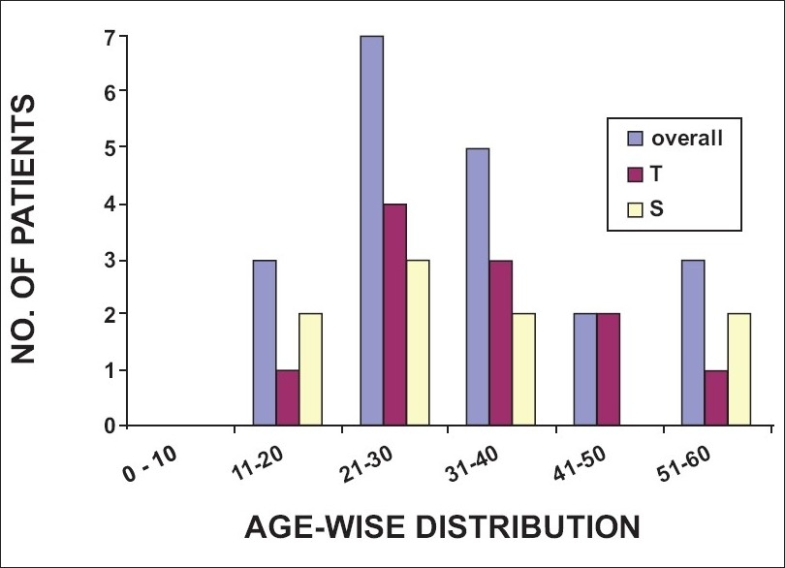
Age distribution of non-survivors

**Figure 3:**
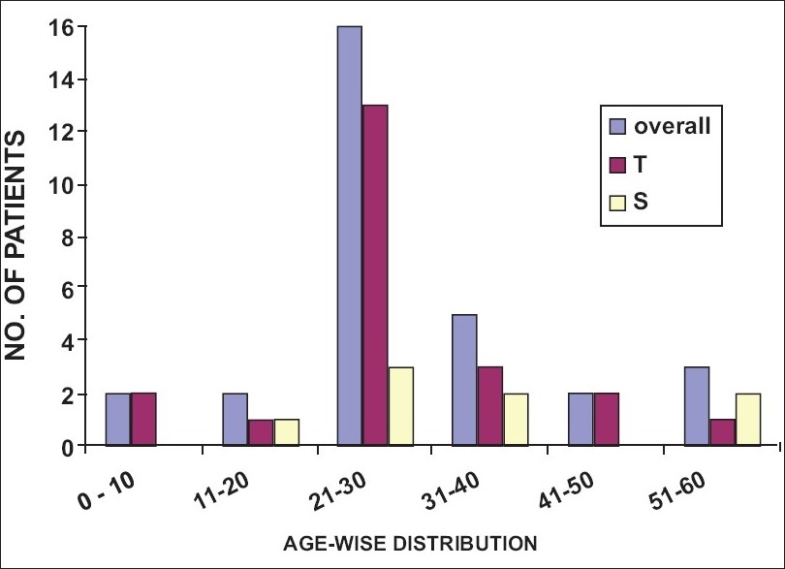
Age distribution of survivors

Of the 50 patients enrolled in the study, 33 were patients of trauma and 17 had ongoing sepsis including SIRS, septic shock, or MODS [Figure 1]. Most patients were between the ages of 21 to 30 years in all four categories. About one-third of the patients of trauma and sepsis non survivors (T/NS and S/NS) (36.4 and 33.3%, respectively) and sepsis survivors (S/S) (37.5%) were of this age group. Two-thirds of the patients in the trauma survivor group (T/S) (59%) were between the ages of 21 to 30 years old [Figures 2 and 3].

An analysis of the lactate values as a quantum in the survivors and non survivors of trauma and sepsis shows a higher set of values in non survivors. Patients of trauma had higher values than patients of sepsis. The range of values obtained remained similar in all the four groups. This clearly shows that a set of lactate values is a more valuable marker than a single value [Table 1].

**Table 1:** Lactate values in non survivors and survivors of sepsis and trauma

**Sepsis/Non Survivors**	
Lactate value	No
<20	14
20-40	19(58.8%)
>40	3(8%)
Total=36 (9 patients) Lactate range 11.2-82.4	

**Sepsis/Survivors**	

Lactate value	No
<20	20
20-40	7
>40	5
Total=32 (8 patients) Lactate range 7.5-72	

**Trauma/Non Survivors**	

Lactate value	No
<20	8
20-40	12(27%)
>40	24(54%)
Total=44 (11 patients) Lactate range 7.5-88.1	

**Trauma/Survivors**	

Lactate value	No
<20	58
20-40	20
>40	10
Total=88 (22 patients) Lactate range 8.4-87.7	

**Figure 4:**
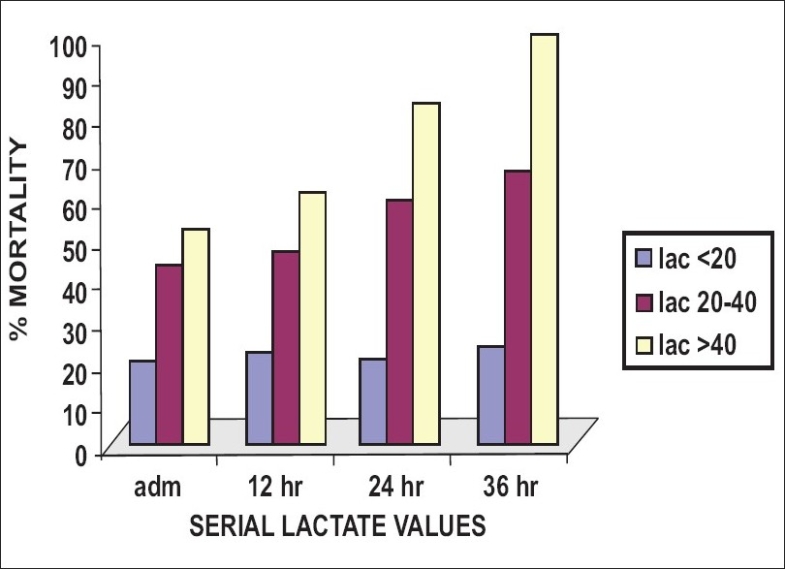
Comparative mortality associated with serial lactate values

As evident from the data, the mean lactate values clearly showed a decreasing trend to normal in trauma survivors as compared with the non survivors. The trauma survivors not only had an obviously lower set of mean lactate values as compared with non survivors but also reached normal levels within 24 hrs in the present study. A similar clear trend was not seen in the sepsis subgroup. However, the sepsis survivors did show a lower range of mean lactate values and the lactate values reached normal levels within 36 hrs. Sepsis non survivors did not ever reach a normal lactate value during the course of follow-up in the study (Table 2a). These findings correlated with studies undertaken by Stacpoole, *et al.*[5] Bakker, *et al.*[6] Pradnya, *et al.*[7] and Meregalli, *et al*.[8] These observations underline the importance of following the trend of lactate levels in critically ill patients. The mean lactate values of patients of trauma and sepsis subgroups combined highlight this trend (a *p*-value <0.05 at 24 and 36 hours of admission; (Table 2b). The predicted mortality rates by the serial lactate levels (for lactate levels >40 mg/dl) at admission, 12, 24, and 36 hours were 52.6%, 61.5%, 83.3%, and 100% respectively for both the trauma and sepsis subgroups combined [Figure 4].

**Table 2a:** Comparative Mean Lactate Values in Various Sub Groups

	Trauma/ non survivors	Trauma/ survivors	Sepsis/ non survivors	Sepsis/ survivors
At Adm	53.04545	35.01364	34.16667	24.375
At 12 hrs	45.90909	23.99091	21.2	30.6125
At 24 hrs	36.50909	14.95455	30.6	22.425
At 36 hrs	32.19091	14.34091	24.44444	17.9375

**Table 2b:** Comparative mean lactate values in trauma and sepsis patients combined

Mean lactate values	Non survivors (trauma +sepsis)	Survivors (trauma +sepsis)	P-value (t test)
At Adm	44.55	32.18	0.643
At 12 hrs	34.790	25.757	0.761
At 24 hrs	33.85	16.95	0.009
At 36 hrs	29.21	15.30	0.000

**Table 2c:** Comparison (of P values) between all variables of non survivors vs. survivors in trauma and sepsis

Trauma Patients: Non Survivors *vs.* Survivors									

T Test									Repeated Measure ANOVA Lactate (P value)
P value	Temperature	Heart rate	Resp rate	MAP	CVP	SpO2	Hrly urine	Lactate

Value 1	0.421969	0.4293842	0.246933	0.3990178	---	0.578768	0.545975	0.041494	0.000
Value 2	0.404181	0.6140392	0.308911	0.2258427	---	0.167396	0.331185	0.001651	
Value 3	0.048242	0.0061413	0.892773	0.0795214	---	0.295198	0.210232	0.002427	
Value 4	0.095362	0.0195599	0.671182	0.0964636	---	0.597569	0.108317	0.017615	

**Sepsis patients: Non survivors vs Survivors**									

T Test									Repeated Measure ANOVA Lactate (P value)

P value	Temperature	Heart rate	Resp rate	MAP	CVP	SpO2	Hrly urine	Lactate

Value 1	0.04807	0.8963791	0.463997	0.2338345	0.48702	0.370465	0.248531	0.300482	0.547
Value 2	0.367837	0.683876	0.697559	0.3177709	0.893419	0.158582	0.453928	0.343714	
Value 3	0.343716	0.4877741	0.196684	0.5720915	0.259992	0.217959	0.774664	0.313782	
Value 4	0.808517	0.2264083	0.189733	0.6781984	0.036567	0.3469	0.759404	0.208658	

It is seen from the table of patient characteristics at baseline and 24 hours that a higher mean level of heart rate (HR) existed in the patients of trauma and sepsis who were non survivors (Table 3; *P*<0.05 at 24 and 36 hours for trauma survivors - the rest were insignificant in all groups; Table 2c); lower MAP and higher mean urinary output in non survivors than survivors (Table 3; *P*-value was insignificant in all groups; Table 2c). However, the mean lactate values were clearly lower in the group of survivors as compared with the non survivors (*p* < 0.05 in trauma subgroup, P insignificant in sepsis subgroup; Table 2c). In general, the patients with sepsis have greater tachycardia, slightly lower MAP, higher CVP, lower urine output, and much lower lactate values than the trauma patients on admission. The difference in the mean heart rate, MAP, CVP, and the hourly urine output between the non survivors and survivors increases in patients of trauma as well as sepsis in comparison with the baseline values. All patients in the study had tachypnea (respiratory rate; (Table 3).

**Table 3:** Variables at Admission and 24 hrs in Different Categories (Values as mean ±standard deviation)

Variable	Trauma/ Non Survivor	Trauma / Survivor	Sepsis / Non Survivor	Sepsis / Survivor
	11	22	9	8
Age (yrs)	18--60	3--52	16--56	14--55
	34±12	28±11	32±15	33±14
Sex			
M	4	17	4	6
F	7	5	5	2
At Inclusion				
Temperature	98.5±0.32	98.6±0.14	98.6±0.4	99.8±1.35
Heart Rate	98.8±16.0	93.5±19.7	111±16.9	109.3±36
RR	24±2.20	24.4±6.9	24.8±1.8	24±2.14
MAP	80.3±31.6	92.7±19.4	78.4±15.0	88.5±14.7
CVP	neg	neg	4.3+3.6	3+1.41
SpO2	97.7±1.28	98.1±1.95	98.1±1.21	95.5±5
Hourly urine output	92.3±39.6	74±61.4	78.3±67.4	46.3±40.2
Lactate	53.0±23.1	35.0±20.4	34.2±19.8	24.4±17.9
At 24 Hrs				
Temperature	99.1±0.71	98.6±0.38	110.1±33.2	99±0.61
Heart Rate	112.5±21.6	89.3±15.6	110.9±21.1	99.8±29.6
RR	23.6±3.74	23.8±5.04	24.8±1.04	23.5±2.33
MAP	61.2±36.4	93.9±10.8	94.3±13.1	90.2±13.8
CVP	neg	2.96±3.31	8.9±3.43	5.9±3.79
SpO2	97.5±2.62	98.6±1.50	99.14±1.07	95±6.32
Hourly urine output	105.9±96.8	65.7±37.5	70.8±55.1	68.8±55
Lactate	36.5±17.8	15±5.8	30.6±20	22.4±11.5
MODS			
Impaired Consciousness	6	-	6	1
Paralytic Ileus	7	-	5	1
ARDS	6	2	3	1
Liver Impairment	-	-	2	2
Renal Failure	3	3	6	1
Surgery	3	14	5	5
Ventilator	7	1	4	-
Dialysis	-	-_	2	1

In non-survivors of trauma, only one patient had hypotension (MAP<65 mmhg) and this patient had a high admission serum lactate of 68 mg/dl. Only one patient (#42) had a normal serum lactate level upon admission, which increased subsequently and the patient died after three days. At 24 hours, two non survivors had normal lactate values (7.5 and 13.4); two other non survivors had normal serum lactates at 36 hours only [Figure 5a]. In the survivors of trauma, one patient had hypotension on admission with a serum lactate level of 37.6 mg/dl. At 36 hours, two survivors still had high serum lactate levels of 29 and 30.7 mg/dl. One of these had persistent high lactate levels at 36 hours of 31.7 mg/dl [Figure 5b]. This patient had a prolonged stay of 28 days.

**Figure 5A:**
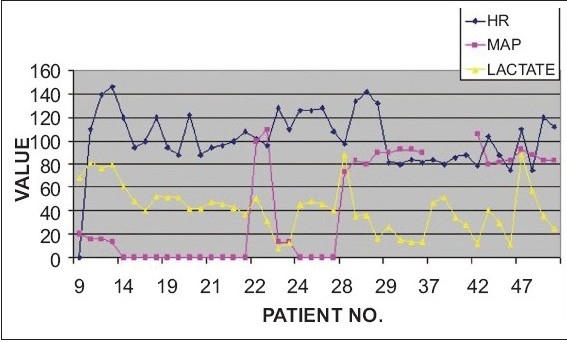
Comparison of Vital Parameters Vs Lactate in Trauma Patients (non survivors)

**Figure 5B:**
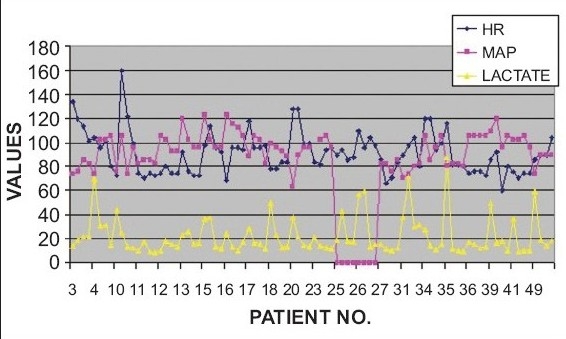
Comparison of Vital Parameters Vs Lactate in Trauma Patients (survivors)

In non survivors of the sepsis subgroup, only one patient had hypotension (MAP 53 mmhg), with a serum lactate level of 26 mg/dl at admission. Four non survivors in the same group had normal lactate values at 24 and 36 hours. Two of these patients (#32, #46) had normal lactate values, throughout the study period. Of these, one patient (#46) showed a rising trend in lactate values and had a hospital stay of 36 days indicating that the patient probably required a longer observation. It is therefore important to infer early from the lactate levels for identifying a potentially ominous trend. In the other patient (#32) whose lactate values remained less than 20 mg/dl, the cause of death eight days later could not be predicted by the lactate levels during the study period [Figure 5c]. Of the survivors of sepsis, one had hypotension at presentation and two had high lactate values of 32.6 mg/dl and 45.3 mg/dl, respectively at 24 hours. The second of the latter had a persistently high lactate level at 36 hours as well (35.4 mg/dl) and had a stay of 48 days. His serial lactate values, however, were on a decreasing trend. Another patient had a marginally high value of 24.3 mg/dl at 36 hours [Figure 5d].

**Figure 5C:**
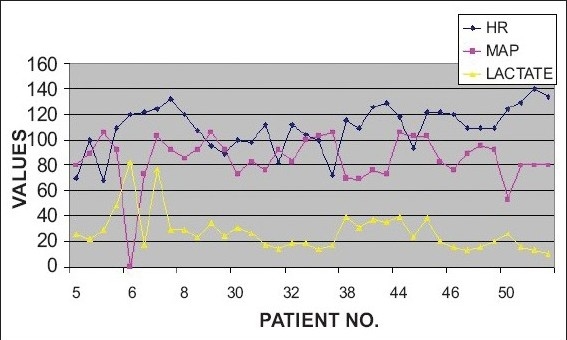
Comparison of Vital Parameters Vs Lactate in Sepsis Patients (non survivors)

**Figure 5D:**
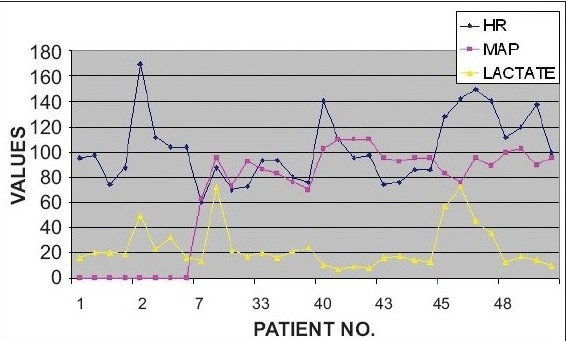
Comparison of Vital Parameters Vs Lactate in Sepsis Patients (survivors)

Patients with extensive burns especially involving extremities who had no accessible sites for measuring blood pressure or oxygen saturation (#1, #2, #14, #19, #21, #24, #25, #26, and # 37) were resuscitated on empirical formulas of burn resuscitation. Invasive arterial pressure monitoring is not a routine practice in our hospital. The inability to monitor resuscitation is thus a disadvantage. The use of the lactate level as a marker of resuscitation will therefore prove to be of worth in this group of patients.[3]

Some survivors of trauma had very high lactate levels (#18, #35, #39, and #49) on admission. Their subsequent lactate levels were normal. In a study, Hogan, *et al*. noted that some patients with only minor injuries have very high lactate levels on admission.[13] The mechanism and location of injury did not significantly dictate mortality or morbidity in this group. They concluded that those patients with associated hypotension or high amounts of blood loss need inpatient admission. Otherwise, discharge within 24 hours may be acceptable when clinically cleared. A very high lactate level upon admission alone does not mandate inpatient admission.

**Figure 6:**
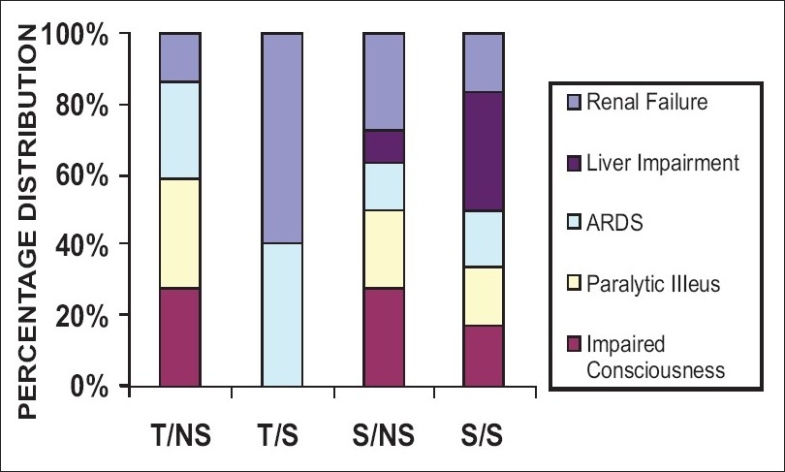
Relative Distribution of Complications (MODS)

Overall, the incidence of manifest organ dysfunction was higher in non survivors than survivors. In the patients of trauma, 10 out of 11 non survivors and 6 out of 22 survivors developed MODS. In the sepsis subgroup, eight out of nine non survivors and four out of eight survivors developed MODS. Only the patients with sepsis showed liver dysfunction [Figure 6]. Ventilator support was required more in the non survivors (7 trauma, 4 sepsis) than the survivors (1 trauma, 0 sepsis). Dialysis was required in three out of 13 patients of renal dysfunction. No mention is made on the surgical intervention due to the difference in the nature and severity of injury in trauma patients. Further, no correlation could be established between the lactate levels and the type or number of organ systems involved in MODS. The APACHE II scoring system is not routinely used in our setup.

## Discussion

Shock, defined as the presence of global tissue hypoxia, is an event that swings the wheel of an ongoing catastrophe in a patient of trauma or sepsis to doom. A relationship between increased blood lactate levels and the presence of tissue hypoxia was suggested as early as 1927 in patients with overt circulatory failure. Many clinical and experimental studies demonstrated that lactate values in blood start to rise when tissue hypoxia is present. As vital parameters are maintained over a wide normal range, this hypoxia may be clinically missed. Thus, while an Intensive Care Unit is abuzz with monitoring of the patient being resuscitated, responding to emergent situations as identified and hemodynamic parameters are attained to within the normal range, the patient may in fact still be in the ‘compensated stage’ of shock. In such patients, serial blood lactate evaluation can be useful to predict shock in the presence of normal physiological parameters and also to predict the development of multiorgan dysfunction as a complication of shock.

Lactate levels are known to be predictors of survival or mortality in patients of trauma and sepsis. A normalization of serum lactate with aggressive treatment within 24 hours of the insult has been shown by others to have a favorable outcome. Aggressive treatment includes timely resuscitation, antibiotics, surgical management, vasopressor and inotropic drugs, ventilatory support, and dialysis as deemed fit. The aim of all interventions remains patient survival. However, the patient presents with a pre-existing set of variables of morbidity that affect his response to an insult and hence the result of these interventions differs between patients.

In a recent publication, Meregalli,*et al.*[8] have shown that despite similar hemodynamic variables, serum lactate values can categorize post-surgical patients into survivors and non survivors within 12 hours of ICU admission. They came to the conclusion that lactate, especially when hemodynamic variables were taken into consideration, seemed to have a similar value in identifying survivors as the SAPS and APACHE II scoring systems and offered even better relevant bedside clinical information in terms of patient condition at the moment.

Porter and Ivatury[9] reviewed the role of traditional endpoints of resuscitation and arrived at the conclusion that using these end points may leave a substantial number of patients, up to 50 to 85% in some cases, in compensated shock, which if allowed to persist would lead to death of the patient. They supported the use of lactate, base deficit, and gastric intramucosal pH as the appropriate endpoints of resuscitation of trauma patients attained within 24 hours of injury for effective resuscitation. The association of lactic acidosis with increased mortality in critically ill patients is well recognized. Lactate levels on admission in trauma patients have been found to correlate with patient survival and also with development of complications. Multiple organ failure after trauma is established within 24 hours of injury in the majority of people who develop it - emphasizing the important role and need for a marker of tissue injury.[10]

In critically ill patients, there is some value in the trend of change in lactate as a means of assessing response to treatment and prognosis. Vincent,*et al.* described the time course of blood lactate in adults who responded to rapid volume resuscitation for circulatory shock and demonstrated the value of serial measurements.[12] In all cases, during the first hour there was at least a 10% reduction in blood lactate. This is in contrast with patients who died during circulatory shock, in whom lactate concentrations did not change with resuscitation. Abramson,*et al.* discussed the results of their study of 76 patients of multi-trauma.[13] They came to the conclusion that hemodynamic optimization of oxygen (O2) delivery, O2 consumption, and cardiac index alone do not predict survival. They clearly showed that the time needed to optimize serum lactate levels is an important prognostic factor for survival in severely injured patients.

Trauma, an ailment afflicting millions worldwide, more commonly the healthy young, prevails as the predominant topic for contributors to research. The value for life triumphs over the value for all material resources. Efforts at all levels manifestly endeavour to maintain this stream of life in the individual afflicted with trauma - to understand the mechanisms involved in the complex chain of events set off in these critical patients, to offer effective resuscitation and supportive treatment, to improve chances of survival, to offer a life that is productive and meaningful. The understanding about various parameters of human physiology that could be monitored to gain an insight into ongoing events in the body has increased over the years. Technology has given a boost to all aspects of medicine. And as the sequence of events following trauma unfolds at the molecular level, the veil is partly lifted over the drama between existence and non existence. The players in the final scene of the drama are possibly the same in the patient who is entangled in the vicious cycle of the syndrome of sepsis. Hence, the search for the signal that will reveal the true state of ongoing events in the body continues to lead to a way that will sustain the stream of life.

Optimization of hemodynamic parameters, as judged by non invasive and also invasive monitoring available may leave a considerable number of patients in compensated shock. Global hypoxia, when allowed to persist, leads to organ dysfunction and death. The lactate level in blood has been known to be a marker of hypoxia. This study was undertaken to see the utility of serial blood lactate as a predictor of shock in our setup in critical patients.

To conclude, serial lactate levels can be used to predict a grave outcome in patients of trauma or sepsis. However, it would be wise to state that the process of recovery from such an insult is a very long one. It can be likened to the war of Troy where many battles were won and lost before the final outcome of the war was declared to the world! Lactate values probably need to be followed for longer periods of time in critical patients even when they have tided over the present crisis. The utility of regular lactate analysis in these patients would depend on factors such as availability and cost of tests as well. There are no existing studies to support the above premise.

Trauma resuscitation that decreases lactate values to normal within 24 hours can be considered effective therapy while increasing levels indicate the need for a more aggressive treatment. They also hint at the need for greater resources for the hospital and the patient.

Patients of sepsis have lower lactate levels in comparison with trauma patients. Interventions that decrease lactate values to normal by as early as 36 hours may improve chances of survival. Traditional parameters of monitoring no doubt have value in following a patient's condition; however, serial lactate values afford a more objective appraisal. Also, in patients with burn injury who have extremity burns and are not invasively monitored, resuscitation can be guided by monitoring lactate levels.
